# Survival Analysis of 4 Different Age Groups of Pancreatic Ductal Adenocarcinoma After Radical Resection From Retrospective Multi‐Center Analysis (YPB‐003)

**DOI:** 10.1002/cam4.70647

**Published:** 2025-02-14

**Authors:** Hiroto Matsui, Tatsuya Ioka, Toru Kawaoka, Tsuyoshi Takahashi, Toshihiro Inokuchi, Eijiro Harada, Kazuhiko Sakamoto, Ryuichiro Suto, Yoshinari Maeda, Taku Nishimura, Yoshitaro Shindo, Yukio Tokumitsu, Masao Nakajima, Yuta Kimura, Taro Takami, Katsuyoshi Ito, Hidekazu Tanaka, Kimikazu Hamano, Hiroaki Nagano, Yamaguchi Pancreatic/Biliary Disease Study Group (YPB)

**Affiliations:** ^1^ Department of Gastroenterological Breast and Endocrine Surgery, Yamaguchi University Graduate School of Medicine Ube Yamaguchi Japan; ^2^ Yamaguchi University Hospital Cancer Center Ube Yamaguchi Japan; ^3^ Department of Surgery Tokuyama Central Hospital Tokuyama Japan; ^4^ Department of Surgery Saiseikai Yamaguchi General Hospital Yamaguchi Japan; ^5^ Department of Surgery Yamaguchi Rosai Hospital Sanyo‐Onoda Japan; ^6^ Department of Surgery and Clinical Science Yamaguchi University Graduate School of Medicine Ube Japan; ^7^ Department of Surgery Kanmon Medical Center Shimonoseki Japan; ^8^ Department of Surgery Yamaguchi Prefectural Grand Medical Center Hofu Japan; ^9^ Department of Surgery Tsushimi Hospital Hagi Japan; ^10^ Department of Gastroenterological Surgery JCHO Shimonoseki Medical Center Shimonoseki Japan; ^11^ Department of Gastroenterology and Hepatology Yamaguchi University Graduate School of Medicine Ube Japan; ^12^ Department of Radiology Yamaguchi University Graduate School of Medicine Ube Japan; ^13^ Department of Radiation Oncology Yamaguchi University Graduate School of Medicine Ube Japan

**Keywords:** adjuvant chemotherapy, frailty, geriatric oncology, pancreatic ductal adenocarcinoma, radial resection

## Abstract

**Aim:**

This study aimed to investigate the efficacy of radical resection and postoperative adjuvant chemotherapy on the survival benefit in patients with pancreatic ductal adenocarcinoma (PDAC), stratified by age, frailty, and other factors in actual clinical practice.

**Methods:**

We retrospectively analyzed the clinicopathological and follow‐up data of 414 patients with PDAC who underwent surgical resection at nine institutions under the Yamaguchi Pancreat/Biliary Disease Study Group, between January 1997 and December 2016. Recurrence‐free survival (RFS) and overall survival (OS) were calculated using the Kaplan–Meier method. Associations between survival and prognostic factors were evaluated by univariate and multivariate analyses.

**Results:**

There were 30.5% of patients with PDAC who were aged < 65 years, 37.9% aged 65–74 years, 17.6% aged 75–79 years, and 14.0% aged ≥ 80 years. Notably, RFS declined with increasing age (median RFS: 12.9, 10.2, 9.4, and 7.4 months, respectively), although the differences were not significant (*p* = 0.223). OS significantly decreased with age (median OS: 21.6, 21.2, 17.0, and 13.9 months, respectively; *p* = 0.005). In patients aged < 75 years, independent prognostic factors identified by univariate and multivariate analyses included lymph node metastasis (hazard ratio [HR], 1.598; *p* = 0.007), tumor size (HR, 1.489; *p* = 0.043), R status (HR, 1.536; *p* = 0.011), and serum albumin levels (HR, 1.526; *p* = 0.031). In patients aged ≥ 75 years, a high modified frailty index (HR, 2.446; *p* = 0.012) emerged as an independent prognostic factor, along with lymph node metastasis, CA19‐9 level (HR, 1.897; *p* = 0.017), and R status (HR, 2.087; *p* = 0.007).

**Conclusion:**

The prognosis for older patients with PDAC was shorter than that of younger patients. Frailty may contribute to their poorer prognosis in older age.

## Graphical abstract

This study aimed to investigate the efficacy of radical resection and postoperative adjuvant chemotherapy on the survival benefit in patients with pancreatic ductal adenocarcinoma, stratified by age, frailty, and other factors in actual clinical practice. High modified frailty index was an independent prognostic factor, in addition to lymph node metastasis and CA19‐9 levels, among those aged > 75 years. Frailty may be strongly related to the prognosis of elderly patients with pancreatic ductal carcinoma after radical resection.

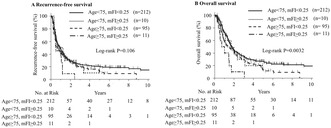

## 1 Introduction

Pancreatic ductal adenocarcinoma (PDAC) is a common gastrointestinal malignancy. PDAC is the 4th leading cause of cancer‐related mortality in both men and women, and its incidence continues to increase at an alarming rate. While surgical resection remains the only potentially curative option, the prognosis after surgery alone, even for T1 PDAC, remains poor [1]. Hence, many clinical trials have been conducted, and adjuvant chemotherapy has been shown to improve the prognosis after radical resection of PDAC [2, 3, 4, 5]. Although clinical trials evaluating the effects of adjuvant chemotherapy tend to favor younger and fit patients, actual clinical practice often involves older and more vulnerable patients. Therefore, it is sometimes difficult to directly apply the results of research in clinical practice.

Yamaguchi, a rural prefecture in western Japan, has a population of approximately 1.3 million spread across 6112 km^2^. While the average aging rate in Japan in 2020 was 28.5%, the aging rate in Yamaguchi Prefecture was 34.3%, which is the third highest in Japan [6]. The aging rate in the Yamaguchi Prefecture may show the situation in Japan 20 years from now. Additionally, rural areas such as Yamaguchi Prefecture are likely to have more vulnerable patients than urban areas [7]. As these vulnerable patients remain underrepresented in clinical trials, the impact of resection and adjuvant chemotherapy for PDAC on this specific population remains unclear.

The goal of this study was to investigate the efficacy of radical resection and postoperative adjuvant chemotherapy on the survival benefit in patients with PDAC stratified by age, frailty, and other factors in Yamaguchi Prefecture.

## 2 Methods

This study was a retrospective data analysis of patients who underwent radical resection for PDAC at nine institutions belonging to the Yamaguchi Pancreat/Biliary Disease Study Group, including two academic centers and seven general hospitals, between January 1997 and December 2016 (Table S1).

### 2.1 Data Collection

Electronic case report forms (eCRFs) were used to collect anonymized demographic and clinical data from the patient charts. Each patient was assigned a unique number as an identifier for the eCRFs. Data related to the demographic profile, tumor details, pathological assessments, treatment, and follow‐up information were collected.

The Eastern Cooperative Oncology Group (ECOG) scale was used to assess performance status at the index date. PDAC was staged according to the 8th Edition of the Union for International Cancer Control (UICC) TNM staging system for PDAC, and cases lacking survival data in the eCRFs were excluded from our analysis. Preoperative blood biochemical test results were collected from the date closest to the surgery within 1 month prior to the procedure. Relative dose intensity (RDI) was calculated using the Hryniuk method [8].

The modified frailty index (mFI) was calculated using 11 distinct elements, as detailed in Table S2. Each element was weighted equally, contributing one point to the index calculation. The mFI was determined by dividing the number of elements present in a patient by the total number of elements [9, 10].

### 2.2 Eligibility Criteria and Exclusion Criteria

**FIGURE 1:**
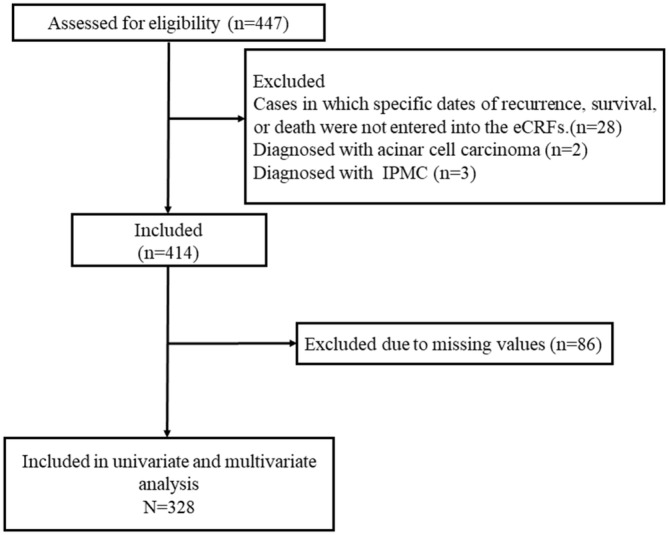
Flow diagram. The flow of study participants through the study.

Eligibility criteria included patients who had invasive pancreatic cancer and had undergone curative resection. The exclusion criteria encompassed primary pancreatic malignancies other than invasive ductal adenocarcinoma and cases in which specific dates of recurrence, survival, or death were not entered into the eCRFs. For the analysis of risk factors for overall survival (OS) using univariate and multivariate methods, cases with “unknown” data were treated as having missing data and were excluded from the analysis. Figure 1 presents a flowchart outlining the patient selection process.

### 2.3 Statistical Analysis

The demographic and tumor characteristics of the patients were summarized using descriptive statistics. Categorical variables were analyzed using Pearson's chi‐square test, and continuous variables were analyzed using a two‐sample *t*‐test. OS was defined as the time from surgery to death from any cause. Survival curves were estimated using the Kaplan–Meier method, and between‐group differences were analyzed using the log‐rank test. A Cox proportional hazards model was used for univariate and multivariate analyses to determine variables associated with OS. The proportional hazards assumptions of the Cox model were assessed using Schoenfeld residual tests.

Data were analyzed using the JMP version 15 software (SAS Institute, Cary, NC, USA). Statistical significance was set at *p* < 0.05. Schoenfeld residuals were analyzed using EZR statistical software, version 1.66 [11, 12].

## 3 Results

### 3.1 Clinicopathological Characteristics

A total of 414 patients who underwent radical resection of PDAC between 1997 and 2016 were included in this study. The overall median follow‐up was 18.4 months (interquartile range, 9.4–40.5 months). Among the participants, 220 (53.1%) were male, and 194 (46.9%) were female, with a median age of 71 years (range: 40–88 years) (Table 1). In terms of age, 30.5% of patients with PDAC were aged < 65 years (young group), 37.9% aged 65–74 years (pre‐old group), 17.6% aged 75–79 years (old group), and 14.0% aged ≥ 80 years (octogenarians group).

**TABLE 1:** Patient characteristics by age group.

	All cases	Young	Pre‐old	Old	Octogenarians	*p*
Number of patients (%)	414	126 (30.5%)	157 (37.9%)	73 (17.6%)	58 (14.0%)	
Age, median (range), years	71 (40–88)	60 (40–64)	70 (65–74)	77 (75–79)	82 (80–88)	< 0.0001*
Gender						
Male	220 (53.1%)	69 (54.8%)	80 (51.0%)	41 (56.2%)	30 (51.7%)	0.862
Female	194 (46.9%)	57 (45.2%)	77 (49.0%)	32 (43.8%)	28 (48.3%)	
Performance status						
0	249 (60.2%)	91 (72.2%)	103 (65.6%)	32 (43.8%)	23 (39.7%)	0.0002*
1	138 (33.3%)	27 (21.4%)	44 (28.0%)	36 (49.3%)	31 (53.5%)	
2	7 (1.7%)	2 (1.6%)	2 (1.3%)	1 (1.4%)	2 (3.4%)	
Unknown	20 (4.8%)	6 (4.8%)	8 (5.1%)	4 (5.5%)	2 (3.4%)	
Body mass index in kg/m^2^, *n* (%)						
< 18.5	72 (17.4%)	17 (13.5%)	26 (16.6%)	19 (26.0%)	10 (17.2%)	0.420
18.5–24.9	269 (65.0%)	82 (65.1%)	105 (66.9%)	40 (54.8%)	42 (72.4%)	
> 25	47 (11.3%)	18 (14.3%)	16 (10.2%)	9 (12.3%)	4 (6.9%)	
Unknown	26 (6.3%)	9 (7.1%)	10 (6.4%)	5 (6.9%)	2 (3.5%)	
Preoperative albumin level (g/dL)						
< 3.5	91 (22.0%)	23 (18.3%)	29 (18.5%)	22 (30.1%)	17 (29.3%)	0.213
≥ 3.5	322 (77.8%)	103 (81.7%)	127 (80.9%)	51 (69.9%)	41 (70.7%)	
Unknown	1 (0.2%)	0 (0%)	1 (0.6%)	0 (0%)	0 (0%)	
Creatinine clearance (Cockcroft‐Gault Formula) (mL/min)						
< 60	134 (32.4%%)	11 (8.7%)	38 (24.2%)	38 (52.1%)	47 (81.0%)	< 0.0001
≥ 60	260 (62.8%)	110 (87.3%)	110 (70.1%)	31 (42.5%)	9 (15.5%)	
Unknown	20 (4.8%)	5 (4.0%)	9 (5.7%)	4 (5.4%)	2 (3.5%)	
Modified frailty index						
< 0.25	319 (77.0%)	102 (81.0%)	121 (77.1%)	52 (71.2%)	44 (75.8%)	0.111
≥ 0.25	21 (5.1%)	1 (0.8%)	9 (5.7%)	8 (11.0%))	3 (5.2%)	
Unknown	74 (17.9%)	23 (18.2%)	27 (17.2%)	13 (17.8%)	11 (19.0%)	
Modified glasgow prognostic score						
0	270 (65.2%)	85 (67.5%)	106 (67.5%)	46 (63.0%)	33 (56.9%)	0.627
1, 2	143 (34.6%)	41 (32.5%)	50 (31.9%)	27 (37.0%)	25 (43.1%)	
Unknown	1 (0.2%)	0 (0%)	1 (0.6%)	0 (0%)	0 (0%)	
Neutrophil‐lymphocyte ratio						
< 5	383 (92.5%)	118 (93.7%)	144 (91.7%)	68 (93.1%)	53 (91.4%)	0.831
≥ 5	29 (7.0%)	8 (6.3%)	12 (7.7%)	4 (5.5%)	5 (8.6%)	
Unknown	2 (0.5%)	0 (0%)	1 (0.6%)	1 (1.4%)	0 (0%	
Preoperative CA19‐9 (units/mL)						
< 37	118 (28.5%)	35 (27.8%)	45 (28.7%)	21 (28.8%)	17 (29.3%)	0.917
≥ 37	289 (69.8%)	89 (70.6%)	108 (68.8%)	52 (71.2%)	40 (69.0%)	
Unknown	7 (1.7%)	2 (1.6%)	4 (2.5%)	0 (0%)	1 (1.7%)	
Neoadjuvant chemotherapy						
No	400 (96.6%)	124 (98.4%)	147 (93.6%)	71 (97.3%)	58 (100%)	0.054
Yes	14 (33.8%)	2 (1.6%)	10 (6.4%)	2 (2.7%)	0 (0%)	
Surgical procedure						
PD	278 (67.2%)	88 (69.8%)	107 (68.2%)	45 (61.6%)	38 (65.5%)	0.518
DP	125 (30.2%)	33 (26.2%)	47 (29.9%)	25 (34.3%)	20 (34.5%)	
TP	11 (26.6%)	5 (4.0%)	3 (1.9%)	3 (4.1%)	0 (0%)	
T status						
T1/T2	324 (78.3%)	98 (77.8%)	123 (78.3%)	60 (82.2%)	43 (74.1%)	0.739
T3/T4	90 (21.7%)	28 (22.2%)	34 (21.7%)	13 (17.8%)	15 (25.9%)	
N status						
Negative	181 (43.7%)	44 (34.9%)	76 (48.4%)	34 (46.6%)	27 (46.6%)	0.122
Positive	233 (56.3%)	82 (65.1%)	81 (51.6%)	39 (53.4%)	31 (53.5%)	
Stage (UICC)						
IA	48 (11.6%)	10 (7.9%)	16 (10.1%)	10 (13.9%)	12 (20.7%)	
IB	95 (22.9%)	22 (17.5%)	42 (26.6%)	21 (29.2%)	10 (17.2%)	
IIA	19 (4.6%)	5 (4.0%)	11 (7.0%)	1 (1.4%)	2 (3.5%)	0.089
IIB	154 (37.2%)	53 (42.1%)	53 (33.5%)	28 (38.8%)	20 (34.5%)	
III	98 (23.7%)	36 (28.5%)	36 (22.8%)	12 (16.7%)	14 (24.1%)	
Residual tumor						
R0	294 (71.0%%)	85 (67.5%)	116 (73.9%)	49 (67.1%)	44 (75.9%)	
R1	99 (23.9%)	33 (26.1%)	33 (21.0%)	21 (28.8%)	12 (20.7%)	
R2	9 (2.2%)	4 (3.2%)	5 (3.2%)	0 (0%)	0 (0%)	0.318
RX	12 (2.9%)	4 (3.2%)	3 (1.9%)	3 (4.1%)	2 (3.5%)	
Unknown	0 (0%)	0 (0%)	0 (0%)	0 (0%)	0 (0%)	
Surgical complication						
CD<III	345 (83.3%)	111 (88.1%)	127 (80.9%)	60 (82.2%)	47 (81.0%)	
CD > =III	53 (12.8%)	12 (9.5%)	23 (14.6%)	9 (12.3%)	9 (15.5%)	0.716
Unknown	16 (3.9%)	3 (2.4%)	7 (4.5%)	4 (5.5%)	2 (3.5%)	
In‐hospital mortality						
No	405 (97.8%)	123 (97.6%)	156 (99.4%)	72 (98.6%)	54 (93.1%)	0.045
Yes	9 (2.2%)	3 (2.4%)	1 (0.6%)	1 (1.4%)	4 (6.9%)	
Adjuvant chemotherapy						
No	143 (34.5%)	35 (27.8%)	42 (26.8%)	33 (45.2%)	33 (56.9%)	
Yes	258 (62.3%	87 (69.1%)	110 (70.0%)	39 (53.4%)	22 (37.9%)	0.0003
Unknown	13 (3.2%)	4 (3.1%)	5 (3.2%)	(1.4%)	3 (5.2%)	
Discontinuation of adjuvant chemotherapy (*n* = 258)						
No	110 (42.6%)	42 (48.3%)	46 (41.8%)	15 (38.4%)	7 (31.8%)	
Yes	130 (50.4%)	41 (47.1%)	56 (50.9%)	20 (51.3%)	13 (59.1%)	0.755
Unknown	18 (7.0)%	4 (4.6%)	8 (7.3%)	4 (10.3%)	2 (9.1%)	
Relative dose intensity of adjuvant chemotherapy, median (range), %						
	65.8 (9.0–100)	77.4 (37.3–100)	65.2 (15.9–100)	55.6 (16.2–100)	63.0 (9.0–100)	0.0025

Abbreviations: CA19‐9, carbohydrate antigen 19–9; CD, Clavien‐Dindo classification; DP, distal pancreatectomy; PD, pancreatoduodenectomy; TP, total pancreatectomy.

*p< 0.05.

Performance status significantly worsened with increasing age. Creatinine clearance decreased with increasing age. No differences in the mFI [13], modified Glasgow prognostic score (mGPS) [14], or neutrophil‐lymphocyte (NL) ratio [15] were observed among the four age groups. No differences were found among the four groups with regard to tumor factors, such as CA19‐9 level, T factor, and N factor. There were no differences in the surgical procedure, R0 ratio, or grade 3 or higher complications, but in‐hospital mortality was significantly higher in octogenarians.

Of all the patients, 62.3% received adjuvant chemotherapy with gemcitabine or S‐1. The proportion of patients who underwent adjuvant chemotherapy decreased with age (young group: 87cases (69.1%), pre‐old group: 110 cases (70.0%), old group: 39 cases (53.4%), octogenerians: 22 cases (37.9%), *p* = 0.0003, Table 1). Table S3 illustrates the distribution of postoperative adjuvant chemotherapy across the four age groups at various time points. From January 1997 to December 2006, of 54 patients, 8 (14.8%) received gemcitabine, none received S‐1, 4 (7.4%) received other adjuvant chemotherapies, and 42 (77.8%) did not receive any adjuvant chemotherapy. Between January 2007 and December 2012, out of 133 patients, 81 (60.9%) received gemcitabine, 15 (11.3%) received S‐1, 4 (3.0%) received other adjuvant chemotherapies, and 33 (24.8%) did not receive adjuvant chemotherapy. From January 2013 to December 2016, among 227 patients, 32 (14.1%) received gemcitabine, 108 (47.6%) received S‐1, 9 (4.0%) received other adjuvant chemotherapies, and 78 (34.4%) did not undergo adjuvant chemotherapy. From 1997 to 2006, most patients across all age groups did not receive adjuvant chemotherapy. From 2007 to 2012, the proportion of patients receiving adjuvant chemotherapy decreased with age, with gemcitabine being the most commonly administered regimen. However, there were no statistically significant differences in regimen use by age. Similarly, between 2013 and 2016, the number of patients receiving adjuvant chemotherapy decreased as they aged, with S‐1 being the most commonly administered regimen. Completion rates did not differ among the four groups of patients receiving adjuvant chemotherapy; however, the relative dose intensities were significantly lower in the older group.

### 3.2 Survival Analysis

**FIGURE 2:**
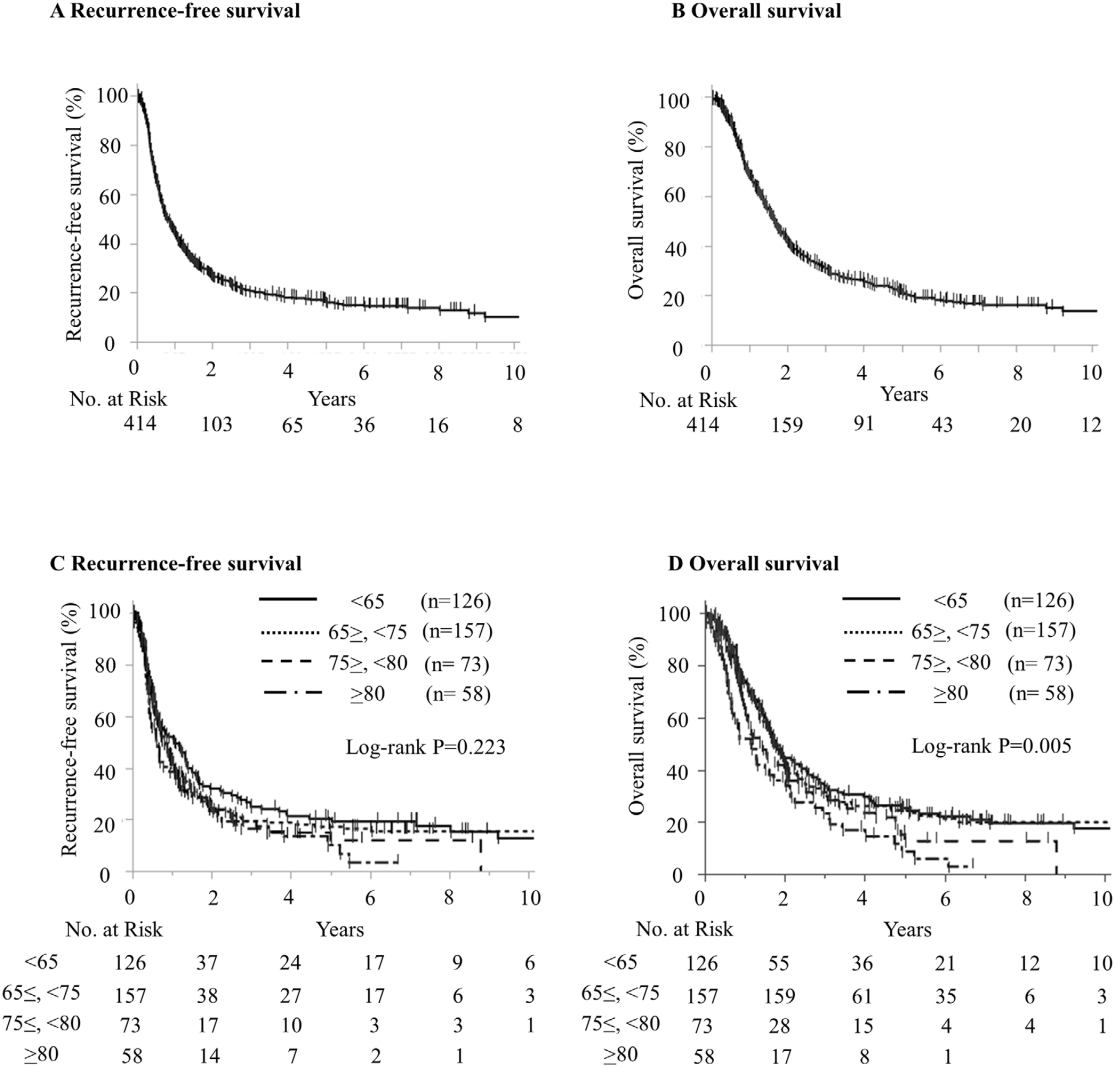
Kaplan–Meier estimates of recurrence‐free survival (RFS) and overall survival (OS). Kaplan–Meier estimates of RFS and OS. (A) RFS in all patients. (B) OS in all patients. (C) RFS according to age. (D) OS by age.

The median recurrence‐free survival (RFS) and OS for all 414 patients were 10.2 and 20.0 months, respectively (Figure 2A,B). The 3‐year RFS and OS rates were 20.5% and 30.8%, respectively; the 5‐year RFS and OS rates were 16.8% and 21.2%, respectively; and the 10‐year RFS and OS rates were 10.2% and 13.6%, respectively. When stratified by age, the median RFS for 126 young patients, 157 pre‐old patients, 73 old patients, and 58 octogenarians was 12.9, 10.2, 9.4, and 7.4 months, respectively. Although there was a trend of decreasing RFS with advancing age, the difference was not significant (*p* = 0.223). In contrast, the median OS for these groups was 21.6, 21.2, 17.0, and 13.9 months, respectively, with a significant decrease observed with advancing age (*p* = 0.005, Figure 2D).

### 3.3 Univariate and Multivariate Survival Analyses

Using the Cox proportional hazards model, we examined the prognostic factors in all patients. A list‐wise deletion approach was used to handle the missing data. Some older patients were censored owing to difficulties in attending hospital visits; however, these cases were retained to ensure the integrity of the OS analysis. A total of 328 patients were included in both univariate and multivariate analyses. Independent prognostic factors identified included R1/R2 resection (hazard ratio [HR]: 1.671, *p* = 0.0002), absence of adjuvant chemotherapy (HR: 1.560, *p* = 0.0017), and lymph node metastasis (HR: 1.918, *p* < 0.0001) (Table 2). Additionally, age ≥ 75 years may be a potential prognostic factor, although there is not a significant difference (HR: 1.321, *p* = 0.075). Schoenfeld residual tests indicated possible violations of the proportional hazards assumption for creatinine clearance, R status, and adjuvant chemotherapy (*p* < 0.05) (Figure S1). However, visual inspection of log–log and Schoenfeld residual plots did not reveal any substantial violations, allowing the analysis to proceed as planned. In addition, because of the lack of difference in oncological background by age, we considered the possibility of differences in prognostic factors between patients over and under 75 years of age. Therefore, we examined the prognostic factors in patients aged > 75 years.

**TABLE 2:** Univariate and multivariate analysis of factors independently associated with overall survival.

Factors	No. patients (%)	Univariate			Multivariate		
	(*n* = 328)	HR	95% CI	*p*	HR	95% CI	*p*
Sex (female)				0.120			
Male	176 (53.7)	1.000	—				
Female	152 (46.3)	1.217	0.950–1.559				
Age				0.0061			0.075
< 75	222 (67.7)	1.000	—		1.000	—	
≥ 75	106 (32.3)	1.439	1.109–1.867		1.321	0.972–1.796	
ECOG performance status				0.067			
0	207 (63.1)	1.000	—				
1/2	121 (36.9)	1.269	0.983–1.639				
Modified Frailty Index				0.056			
< 0.25	307 (93.6)	1.000	—				
≥ 0.25	21 (6.4)	1.598	0.987–2.587				
Albumin				0.005			0.765
≥ 3.5 g/dL	259 (79.0)	1.000	—		1.000	—	
< 3.5 g/dL	69 (21.0)	1.519	1.134–2.034		1.064	0.708–1.598	
Creatinine clearance				0.0047			0.330
≥ 60 mL/min	221 (67.4)	1.000	—		1.000	—	
< 60 mL/min	107 (32.6)	1.458	1.123–1.893		1.164	0.858–1.578	
Modified glasgow prognostic score				0.037			0.607
0/1	293 (89.3)	1.000	—		1.000		
2	35 (10.7)	1.485	0.206–2.050		1.145	0.683–1.920	
Neutrophil‐lymphocyte ratio				0.646			
< 5	308 (93.9)	1.000	—				
≥ 5	20 (6.1)	1.126	0.678–1.871				
Tumor size							0093
< 20 mm	80 (24.5)	1.000	—	0.0093	1.000		
≥ 20 mm	246 (75.5)	1.494	1.104–2.020		1.304	0.957–1.779	
T stage				0.071			
T1–T2	255 (77.7)	1.000	—				
T3–T4	73 (22.3)	1.307	0.977–1.747				
N factor				< 0.0001			< 0.0001
Negative	144 (43.9)	1.000	—		1.000	—	
Positive	184 (56.1)	2.018	1.557–2.615		1.916	1.467–2.504	
Pretreatment CA19‐9				0.0152			0.103
< 37 U/mL	92 (28.0)	1.000	—		1.000		
≥ 37 U/mL	236 (72.0)	1.425	1.071–1.897		1.276	0.952–1.711	
R status				0.0002			0.0002
R0	224 (68.3)	1.000	—		1.000		
R1/R2	104 (31.7)	1.659	1.276–2.157		1.671	1.274–2.190	
Adjuvant chemotherapy				0.0082			0.0017
Yes	209 (63.7)	1.000	—		1.000		
No	119 (36.3)	1.415	1.094–1.829		1.560	1.181–2.062	
Neoadjuvant chemotherapy				0.409			
Yes	14 (4.3)	1.000	—				
No	314 (95.7)	1.373	0.647–2.912				

Abbreviations: CA19‐9, carbohydrate antigen 19–9; CI, confidence interval; ECOG, Eastern Cooperative Oncology Group; HR, hazard ratio.

Specifically, in patients aged < 75 years, independent prognostic factors included lymph node metastasis, tumor size, serum albumin levels, and R status (Table 3), with no violations of the proportional hazards assumption confirmed by Schoenfeld residual tests (Figure S2). By contrast, for patients aged ≥ 75 years, a high mFI and no adjuvant chemotherapy were identified as additional independent prognostic factors, alongside lymph node metastasis, CA19‐9 levels, and R status (Table 4), with no violations of the proportional hazards assumption according to Schoenfeld residual test results (Figure S3).

**TABLE 3:** Univariate and multivariate analysis of factors independently associated with overall survival in PDAC patients under 75 years of age.

Factors	No. patients (%)	Univariate			Multivariate		
	(*n* = 222)	HR	95% CI	*p*	HR	95% CI	*p*
Sex (female)				0.050			0.240
Male	117 (52.7)	1.000	—		1.000		
Female	105 (47.3)	1.359	1.000–1.849		1.212	0.879–1.671	
ECOG performance status				0.753			
0	162 (73.0)	1.000	—				
1/2	60 (27.0)	1.058	0.746–1.500				
Modified frailty index				0.056			
< 0.25	212 (95.5)	1.000	—				
≥ 0.25	10 (4.5)	1.598	0.987–2.587				
Albumin				0.0006			0.031
≥ 3.5 g/dL	182 (82.0)	1.000	—		1.000	—	
< 3.5 g/dL	40 (18.0)	1.912	1.321–2.768		1.526	1.041–2.238	
Creatinine clearance				0.0313			0.058
≥ 60 mL/min	183 (82.4)	1.000	—		1.000	—	
< 60 mL/min	39 (17.6)	1.531	1.039–2.257		1.459	0.987–2.155	
Modified glasgow prognostic score				0.0037			0.322
0/1	201 (90.5)	1.000	—		1.000		
2	21 (9.5)	2.009	1.253–3.219		1.449	0.696–3.017	
Neutrophil‐lymphocyte ratio				0.893			
< 5	208 (93.7)	1.000	—				
≥ 5	14 (6.3)	1.045	0.550–1.983				
Tumor size							0.043
< 20 mm	56 (25.2)	1.000	—	0.0055	1.000		
≥ 20 mm	166 (74.8)	1.669	1.142–2.439		1.489	1.013–2.190	
T stage^a^				0.421			
T1–T2	175 (78.8)	1.000	—				
T3–T4	47 (21.2)	1.164	0.804–1.685				
N factor				< 0.0001			0.007
Negative	96 (43.2)	1.000	—		1.000	—	
Positive	126 (56.8)	1.881	1.365–2.593		1.598	1.140–2.240	
Pretreatment CA19‐9				0.147			
< 37 U/mL	59 (26.6)	1.000	—				
≥ 37 U/mL	163 (73.4)	1.308	0.910–1.881				
R status				0.0063			0.011
R0	151 (68.0)	1.000	—		1.000		
R1/R2	71 (32.0)	1.567	1.136–2.163		1.536	1.105–2.135	
Adjuvant chemotherapy				0.3807			
Yes	162 (73.3)	1.000	—				
No	60 (36.7)	1.171	0.826–1.660				
Neoadjuvant chemotherapy				0.6775			
Yes	12 (5.4)	1.000	—				
No	210 (94.6)	1.184	0.523–2.680				

Abbreviations: CA19‐9, carbohydrate antigen 19–9; CI, confidence interval; ECOG, Eastern Cooperative Oncology Group; HR, hazard ratio; PDAC, pancreatic ductal adenocarcinoma.

^a^According to the eighth Union for International Cancer Control TNM classification.

**TABLE 4:** Univariate and multivariate analysis of factors independently associated with overall survival in PDAC patients aged 75 years and over.

Factors	No. patients (%)	Univariate			Multivariate		
	(*n* = 106)	HR	95% CI	*p*	HR	95% CI	*p*
Sex (female)				0.734			
Male	59 (55.7)	1.000	—				
Female	47 (44.3)	0.929	0.607–1.421				
ECOG performance status				0.090			
0	45 (42.5)	1.000	—				
1/2	61 (57.5)	1.453	0.943–2.241				
Modified frailty index				0.029			0.040
< 0.25	95 (89.6)	1.000	—		1.000	—	
≥ 0.25	11 (10.4)	2.115	1.079–4.144		2.259	1.036–4.924	
Albumin				0.736			
≥ 3.5 g/dL	77 (72.6)	1.000	—				
< 3.5 g/dL	29 (27.4)	0.920	0.566–1.495				
Creatinine clearance				0.973			
≥ 60 mL/min	38 (35.8)	1.000	—				
< 60 mL/min	68 (64.2)	0.993	0.641–1.536				
Modified glasgow prognostic score				0.766			
0/1	92 (86.8)	1.000	—				
2	14 (13.2)	0.911	0.495–1.680				
Neutrophil‐lymphocyte ratio				0.495			
< 5	100 (94.3)	1.000	—				
≥ 5	6 (5.7)	1.337	0.580–3.079				
Tumor size							
< 20 mm	25 (23.6)	1.000	—	0.668			
≥ 20 mm	81 (76.4)	1.116	0.676–1.842				
T stage				0.096			
T1–T2	80 (75.5)	1.000	—				
T3–T4	26 (24.5)	1.507	0.930–2.442				
N factor				< 0.0001			< 0.0001
Negative	48 (45.3)	1.000	—		1.000	—	
Positive	58 (54.7)	2.619	1.665–4.119		2.905	1.765–4.779	
Pretreatment CA19‐9				0.0034			0.017
< 37 U/mL	33 (31.1)	1.000	—		1.000		
≥ 37 U/mL	73 (68.9)	2.052	1.268–3.319		1.897	1.120–3.214	
R status				0.0010			0.007
R0	73 (68.9)	1.000	—		1.000		
R1/R2	33 (31.1)	2.194	1.373–3.507		2.087	1.227–3.549	
Adjuvant chemotherapy				0.036			0.002
Yes	47 (44.3)	1.000	—		1.000		
No	59 (55.7)	1.577	1.031–2.413		2.089	1.309–3.333	
Neoadjuvant chemotherapy				0.460			
Yes	2 (1.9)	1.000	—				
No	104 (98.1)	2.109	0.523–2.680				

Abbreviations: CA19‐9, carbohydrate antigen 19–9; CI, confidence interval; ECOG, Eastern Cooperative Oncology Group; HR, hazard ratio; PDAC, pancreatic ductal adenocarcinoma.

### 3.4 Influence of Age and Frailty on Prognosis

**FIGURE 3:**
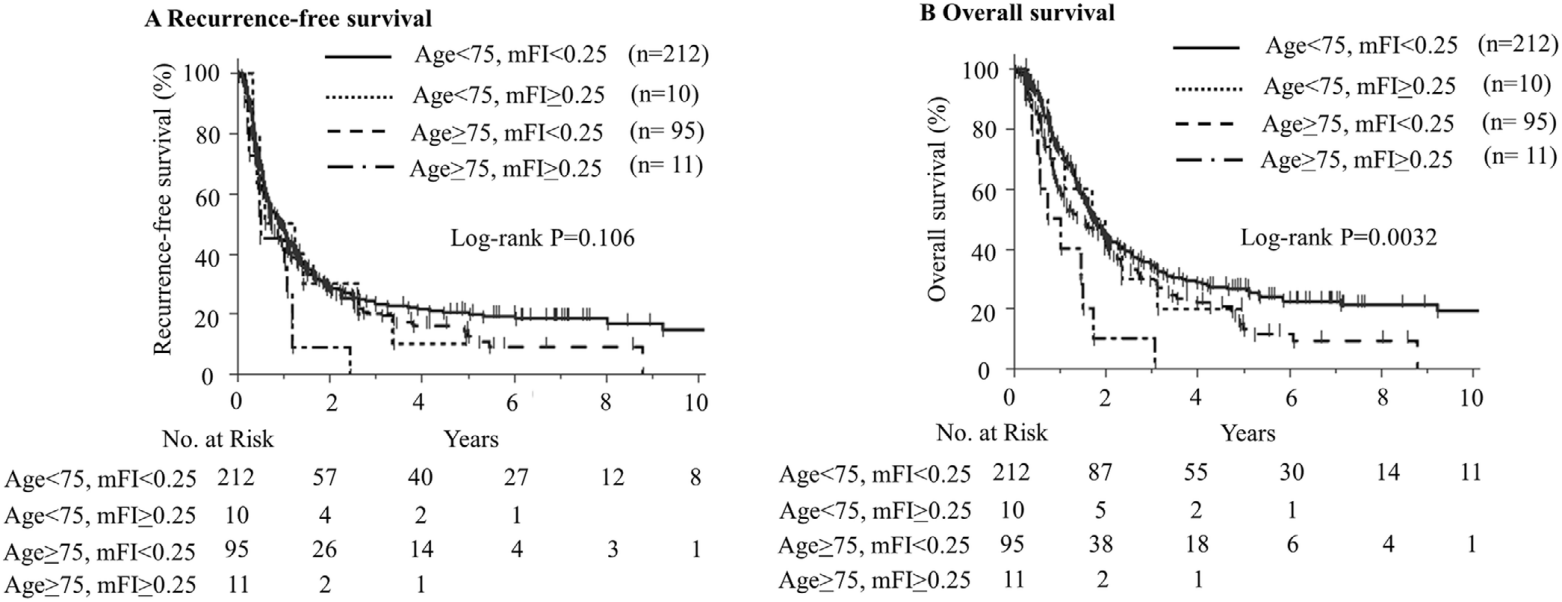
Kaplan–Meier estimates of recurrence‐free survival (RFS) and overall survival (OS) with age and modified frailty index. (A) RFS with age and modified Frailty index. (B) OS with age and modified frailty index (mFI).

The Kaplan–Meier curves, which were divided into four groups according to age and mFI, are shown in Figure 3. The group aged 75 years with a high mFI had significantly poorer OS (*p* = 0.0032, log‐rank test). Figure S4 presents Kaplan–Meier curves for 323 cases, excluding surgery‐related mortality, to mitigate the impact of surgery‐related deaths. These curves similarly indicated that patients > 75 years with higher mFI had a significantly poorer OS prognosis (*p* = 0.003, log‐rank test).

Table S4 presents the background and clinical characteristics of the four groups stratified by age and mFI. In patients aged ≥ 75 with high mFI, there was a significantly higher proportion with a performance status of 1, lower creatinine clearance, a reduced rate of postoperative adjuvant chemotherapy, and among those who received it, a significantly lower RDI.

### 3.5 Survival Outcomes by Adjuvant Chemotherapy and the Impact of Frailty and Age

**FIGURE 4:**
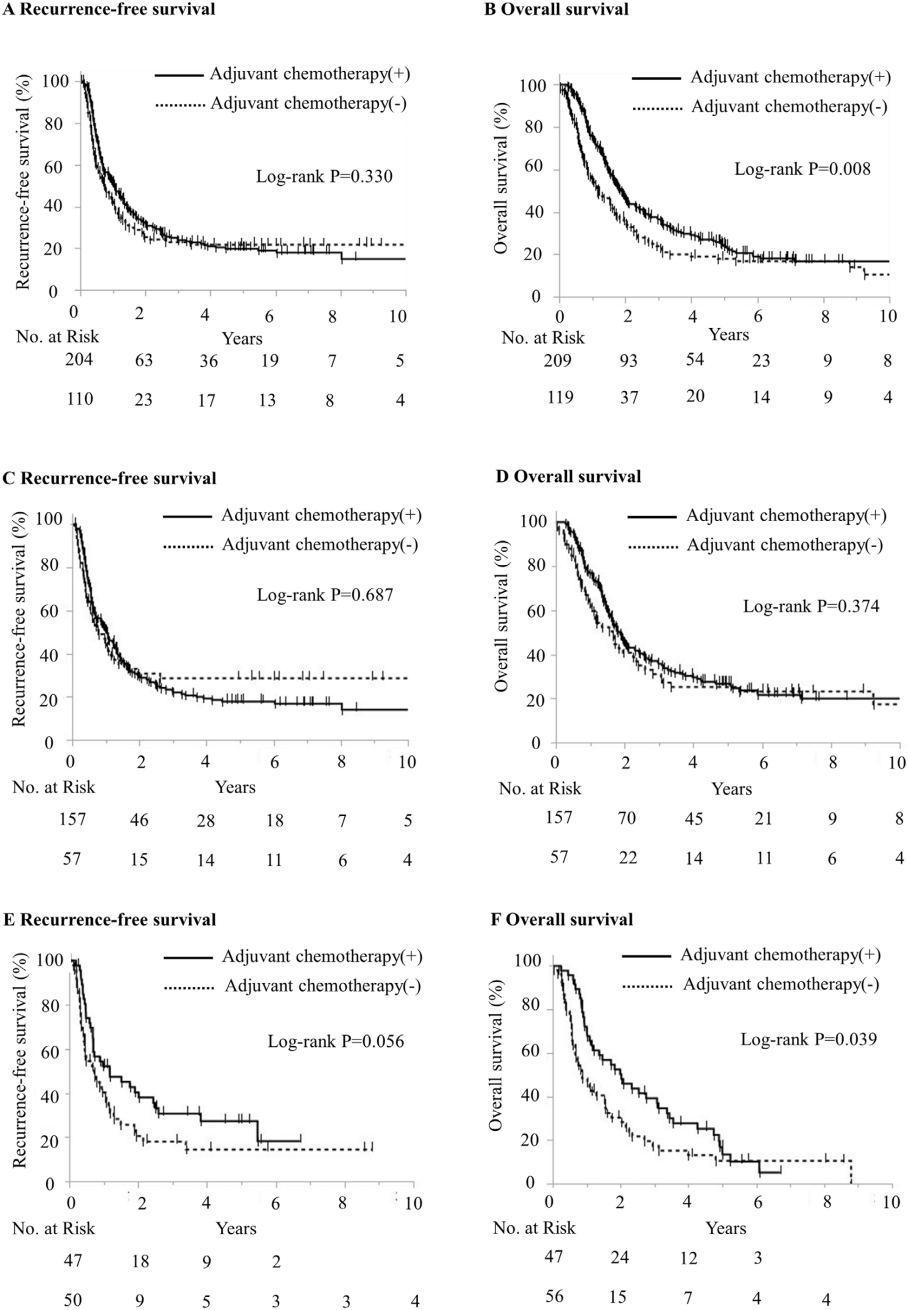
Kaplan–Meier estimates of recurrence‐free survival and overall survival with adjuvant chemotherapy or without chemotherapy. (A) RFS in all patients. (B) OS in all patients. (C) RFS among patients aged < 75 years. (D) OS among patients aged < 75 years. (E) RFS among patients aged ≥ 75 years. (F) OS RFS among patients aged ≥ 75 years.

We employed the Kaplan–Meier method to analyze RFS and OS based on adjuvant chemotherapy administration. The median RFS was 12.6 months in the adjuvant chemotherapy group compared to 9.0 months in the non‐chemotherapy group; however, this difference was not statistically significant (*p* = 0.330) (Figure 4A). By contrast, the median OS was significantly longer in the adjuvant chemotherapy group than in the non‐chemotherapy group (22.6 months vs. 13.8 months, *p* = 0.008) (Figure 4B). Among patients aged < 75 years, the median RFS was 12.5 months for the adjuvant chemotherapy group vs. 9.4 months for the non‐chemotherapy group (*p* = 0.687), and the median OS was 22.5 months versus 20.0 months, respectively, with no significant difference (*p* = 0.374) (Figure 4C,D). Conversely, in patients aged ≥ 75 years, the median RFS tended to be longer in the adjuvant chemotherapy group compared to the non‐chemotherapy group (14.1 months vs. 8.7 months, *p* = 0.056) (Figure 4E). Similarly, the median OS was significantly longer in the adjuvant chemotherapy group compared to the non‐chemotherapy group (22.4 months vs. 10.4 months, *p* = 0.039) (Figure 4F).

**FIGURE 5:**
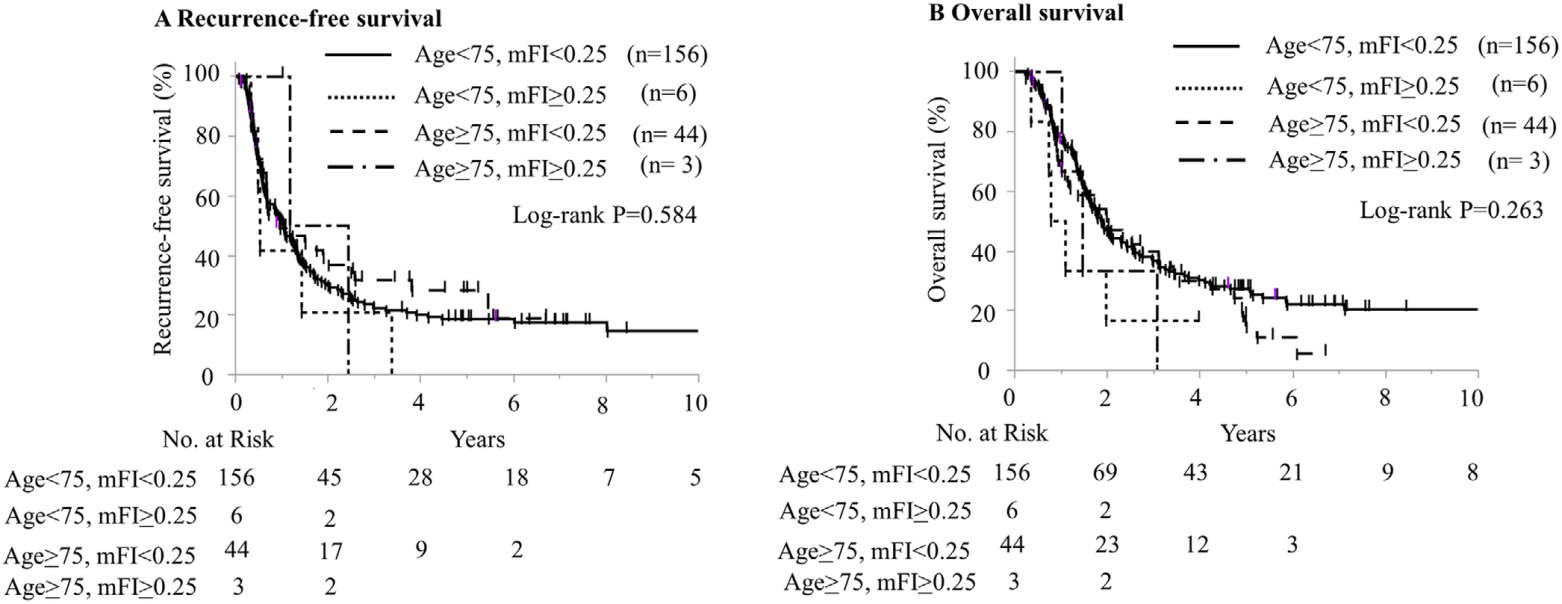
Kaplan–Meier estimates of recurrence‐free survival (RFS) and overall survival (OS) with adjuvant chemotherapy. (A) RFS with age and modified Frailty index. (B) OS with age and modified frailty index (mFI).

To further evaluate the impact of adjuvant chemotherapy, we stratified patients into four groups based on age and mFI. The initiation rates of adjuvant chemotherapy were 73%, 60%, 46%, and 28% for the low and high mFI groups < 75 years and > 75 years, respectively, with a significantly lower initiation rate in the high mFI group aged ≥ 75 years (*p* < 0.001). No significant differences in median RFS were observed among the four groups (Age < 75, mFI < 0.25: 12.7 months; Age < 75, mFI > 0.25: 6.4 months; Age ≥ 75, mFI < 0.25: 11.7 months; Age ≥ 75, mFI > 0.25: 21.8 months; *p* = 0.584) (Figure 5A). The 3‐year and 5‐year RFS rates were 22.1% and 18.4% in the < 75 years, low mFI group; 20.8% and 0% in the < 75 years, high mFI group; 31.6% and 28.7% in the ≥ 75 years, low mFI group; and 0% for both in the ≥ 75 years, high mFI group. Similarly, median OS did not differ significantly among the four groups (Age < 75, mFI < 0.25: 22.6 months; Age < 75, mFI > 0.25: 11.3 months; Age ≥ 75, mFI < 0.25: 24.4 months; Age ≥ 75, mFI > 0.25: 17.7 months; *p* = 0.263) (Figure 5B). The 3‐year and 5‐year OS rates were 36.5% and 27.2% for the < 75 years, low mFI group; 16.7% and not assessable for the < 75 years, high mFI group; 39.6% and 18.0% for the ≥ 75 years, low mFI group; and 0% for both in the ≥ 75 years, high mFI group.

## 4 Discussion

As in previous reports [16, 17], our retrospective observational study showed that older patients who underwent surgical resection for PDAC had a worse prognosis than younger patients. In addition, our study revealed that prognostic factors differed between older and younger patients, and mFI was identified as a prognostic factor in older patients.

It was also observed that OS worsened with age. Because the current study was retrospective, disease‐specific survival rates could not be verified owing to the lack of details on the cause of death in some cases. Furthermore, no differences in oncologic factors such as T factor, lymph node metastasis, and CA19‐9 levels were observed according to age. In terms of perioperative outcomes, octogenarians showed a significantly higher rate of in‐hospital mortality than other age groups. However, the proportion of in‐hospital deaths among octogenarians was small in relation to the total number of cases, which may have had little impact on the RFS and OS. In contrast, no differences were observed in surgical procedures, R0 rates, or grade 3 or higher complications. Therefore, it is unlikely that perioperative factors had a significant impact on the differences in RFS and OS according to age.

It has been reported that OS worsens with age in cases of gastric and colon cancer [18, 19, 20], and is more pronounced in advanced stages of the disease. One reason for this may be that elderly patients are less likely to receive adjuvant chemotherapy after surgery compared to younger patients, and even when they do receive adjuvant chemotherapy, they may not achieve an effective RDI. As shown in Table 1, the induction rate of adjuvant chemotherapy decreased with increasing age. Older patients who received adjuvant chemotherapy had a MST comparable to that of younger patients, while the MST of older patients who did not receive chemotherapy was about half of that. This may have led to a poorer prognosis for the elderly as a whole. PDAC is considered to have strong systemic disease characteristics, even when it is resectable. As previous clinical trials have shown [2, 3], surgery alone results in a poor prognosis, whereas postoperative adjuvant chemotherapy is expected to improve the prognosis. However, despite postoperative adjuvant chemotherapy, patients receiving a low RDI have a poor prognosis [21]. With increasing age, there were more cases in which postoperative adjuvant chemotherapy was not administered (Table 1). Even in cases that underwent chemotherapy, there was a lower RDI in older patients than in younger patients.

From January 1997 to December 2016, significant changes occurred in adjuvant chemotherapy practices, reflecting advancements in this therapeutic approach following pivotal studies [2, 3]. Despite these advancements, a consistent trend was observed in this study where the administration of adjuvant chemotherapy decreased with increasing patient age. Both OS and RFS were significantly longer in patients who received adjuvant chemotherapy than in those who did not (Figure 4). However, owing to the non‐randomized nature of this study, we speculate that considerable selection bias might have likely influenced the group that did not receive adjuvant chemotherapy. While improvements in adjuvant chemotherapy have yielded some prognostic benefits, these gains diminish with age, resulting in limited enhancements for older patients. This persistent phenomenon underscores the challenge of achieving significant prognostic improvements in older patients despite the potential efficacy of adjuvant chemotherapy.

Previous studies have reported on prognostic factors after resection of pancreatic cancer [22, 23, 24, 25, 26, 27]. We conducted both univariate and multivariate analyses to examine clinicopathological factors associated with prognosis. Our findings confirmed that lymph node metastasis, R1/R2 resection, and absence of adjuvant chemotherapy were significant prognostic indicators. Age also demonstrated a tendency to affect prognosis, indicating that prognostic factors may vary among patients of different age groups owing to biological differences. For instance, in patients < 75 years, independent prognostic factors included serum albumin levels < 3.5 g/dL, lymph node metastasis, tumor size, and R1/R2 resection, whereas for patients ≥ 75 years, significant factors included lymph node metastasis, elevated CA19‐9 levels, R1/R2 resection, and a high mFI. Similarly, it has been previously reported that lymph node metastasis and CA19‐9 levels are important prognostic factors after radical resection of PDAC [28, 29, 30, 31, 32, 33].

Several studies have reported that frailty affects prognosis after radical resection of PDAC [34, 35]; thus, we considered frailty as an important factor in this study. However, only a small number of cases were evaluated for frailty at the beginning of the treatment intervention. The mFI consists of 11 comorbidities, which are all included in the American College of Surgeons National Surgical Quality Improvement Program (ACS‐NSQIP) [13] and can be assessed retrospectively from clinical records. Moreover, a relationship between the mFI and prognosis after radical resection has been reported for some malignancies, although few studies have reported such an association in PDAC [36, 37]. Therefore, we used the mFI to assess frailty.

As reported in previous studies, frailty is defined as a clinical state of increased vulnerability, which is induced by an aging‐associated decline in function and reserve across multiple physiological systems [38]. In this study, younger patients might have had fewer differences in organ capacity, such as renal function. As a result, in the perioperative period and postoperative adjuvant chemotherapy, there might have been few differences, and only lymph node metastasis might have been an independent prognostic factor. However, there may be large inter‐individual differences in organ capacity among older individuals. These differences may have an impact on the perioperative course and RDI of postoperative adjuvant chemotherapy. Notably, a substantial proportion of older patients in the high mFI group had a PS of 1 and poorer renal function. These patients were less likely to receive postoperative adjuvant chemotherapy, and when they did, it was administered at a lower RDI. This suggests that reduced renal function and PS influenced both the postoperative adjuvant chemotherapy administration rate and RDI. Although there were no differences in chemotherapy discontinuation rates, this could be attributed to selection bias, as many older patients with high mFI scores did not receive chemotherapy, resulting in a cohort of older chemotherapy recipients who were generally fitter with lower mFI scores. This selection bias may explain the similar discontinuation rates observed across age groups (Table S4). These effects may be reflected in mFI; therefore, mFI may also be an independent prognostic factor in older patients. Notably, the prognosis for patients over 75 years of age with a high mFI score was extremely poor, with an MST of 10.6 months and death occurring within approximately 3 years in all cases. Under current utilization practices, the clinical effectiveness of surgery and adjuvant chemotherapy is limited to patients with PDAC aged 75 years or older with a high mFI. However, patients over 75 years of age with a low mFI might have a similar prognosis as younger patients (Figure 5). These findings suggest that the mFI could indeed be a valuable prognostic indicator for patients with PDAC aged 75 years.

Our study has some limitations. First, this was a retrospective study; thus, to confirm our findings, a multicenter prospective validation study is necessary. Second, the study period was relatively long, and only a few patients received neoadjuvant chemotherapy, which is now considered the standard treatment. However, older patients with high mFI may not have received a sufficient RDI of neoadjuvant chemotherapy, which is more intensive than postoperative adjuvant chemotherapy, if they have received it. The effectiveness of neoadjuvant chemotherapy in older patients with a high mFI should also be assessed in prospective studies. Third, frailty was evaluated retrospectively using the mFI because only a small number of cases were assessed for frailty at the start of the treatment intervention. A prospective comprehensive geriatric assessment is required to further investigate the relationship between frailty and PDAC prognosis. Lastly, the study had a relatively high number of censored cases, primarily owing to incomplete follow‐up data, particularly among older patients who faced challenges in attending hospital visits. Consequently, some patients were lost to follow‐up or transferred to other healthcare facilities, resulting in censoring at their last known follow‐up date. While this was accounted for in the statistical analysis, it may have affected the robustness of our survival estimates.

## 5 Conclusions

In conclusion, this retrospective study revealed that, unlike in younger patients, frailty may be strongly related to the prognosis of PDAC patients after radical resection in older patients.

The mFI can serve as a convenient tool to screen frailty in patients with PDAC before treatment, similar to its use in other cancers. Given PDAC's higher malignancy compared to other cancers, it often requires multidisciplinary treatment, and the mFI may play a key role in tailoring chemotherapy intensity and optimizing individualized patient care.

## Author Contributions

**Hiroto Matsui:** conceptualization (lead), data curation (lead), formal analysis (equal), funding acquisition (equal), investigation (equal), methodology (lead), project administration (lead), resources (equal), software (equal), supervision (equal), validation (equal), visualization (equal), writing – original draft (lead), writing – review and editing (equal). **Tatsuya Ioka:** investigation (equal), project administration (supporting). **Toru Kawaoka:** investigation (equal). **Tsuyoshi Takahashi:** investigation (equal). **Toshihiro Inokuchi:** data curation (equal), investigation (equal). **Eijiro Harada:** data curation (equal), investigation (equal). **Kazuhiko Sakamoto:** data curation (equal), investigation (equal). **Ryuichiro Suto:** data curation (equal), investigation (equal). **Yoshinari Maeda:** data curation (equal), investigation (equal). **Taku Nishimura:** data curation (equal), investigation (equal). **Yoshitaro Shindo:** data curation (equal), investigation (equal). **Yukio Tokumitsu:** data curation (equal), investigation (equal). **Masao Nakajima:** data curation (equal), investigation (equal). **Yuta Kimura:** data curation (equal), investigation (equal). **Taro Takami:** data curation (equal), investigation (equal). **Katsuyoshi Ito:** data curation (equal), investigation (equal). **Hidekazu Tanaka:** data curation (equal), investigation (equal). **Kimikazu Hamano:** data curation (equal), investigation (equal). **Hiroaki Nagano:** data curation (equal), investigation (equal), methodology (equal).

## Ethics Statement

This study was reviewed and approved by the institutional Ethical Board of Yamaguchi University Graduate School of Medicine and Graduate School of Medicine, Osaka University (IRB number: H2019‐041). The need for informed consent was waived by the Ethics Committee/Institutional Review Board of Yamaguchi University Graduate School of Medicine and Graduate School of Medicine, Osaka University due to the retrospective nature of the study. The study was conducted in accordance with Good Clinical Practice guidelines and the Declaration of Helsinki.

## Conflicts of Interest

The authors declare no conflicts of interest.

## Supporting information


**Figure S1.** Schoenfeld residuals used to examine the proportional hazards assumption for the variables included in Cox PH regression model in Table 2.

**Figure S2.** Schoenfeld residuals used to examine the proportional hazards assumption for the variables included in Cox PH regression model in Table 3.

**Figure S3.** Schoenfeld residuals used to examine the proportional hazards assumption for the variables included in Cox PH regression model in Table 4.

**Figure S4.** The Kaplan–Meier curves for 323 cases excluding cases of surgery‐related mortality. Kaplan–Meier estimates of recurrence‐free survival (RFS) and overall survival (OS). (A) RFS in all patients. (B) OS in all patients. (C) RFS according to age. (D) OS by age.

**Table S1.** Participating institutions and number of cases.

**Table S2.** 11 components of the modified frailty index.

**Table S3.** Adjuvant Chemotherapy in Four Age Groups at Different Time Points.

**Table S4.** Modified frailty index and organ function.

## Data Availability

The datasets used and/or analyzed during the current study are available from the corresponding author (HN).
