# Supplementary figures and images for: Survival Analysis of 4 Different Age Groups of Pancreatic Ductal Adenocarcinoma After Radical Resection From Retrospective Multi‐Center Analysis (YPB‐003)
Source: Cancer Med. 2025 Feb 14;14(4):e70647. doi: 10.1002/cam4.70647 (PMC11826832; doi:10.1002/cam4.70647)

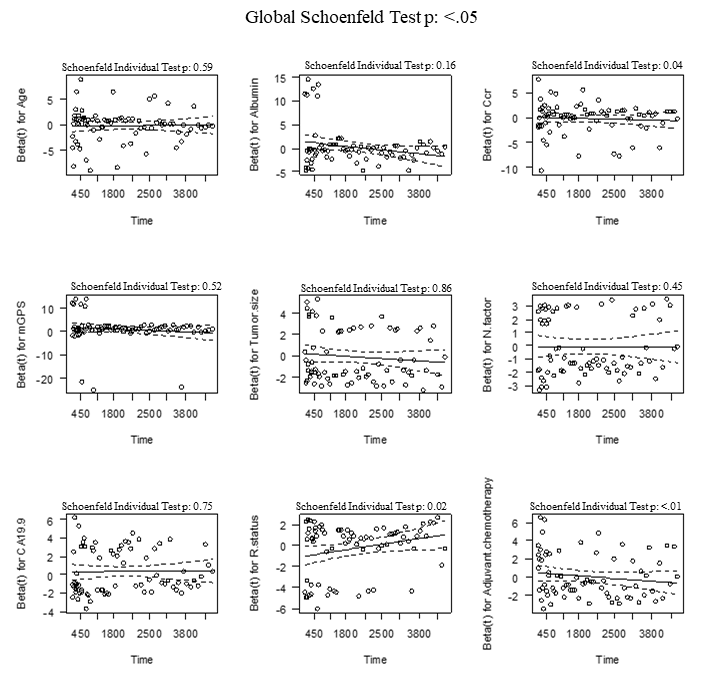

Supplement: Supplementary file 1 — Figure S1. Schoenfeld residuals used to examine the proportional hazards assumption for the variables included in Cox PH regression model in Table 2. [file CAM4-14-e70647-s005.tif]

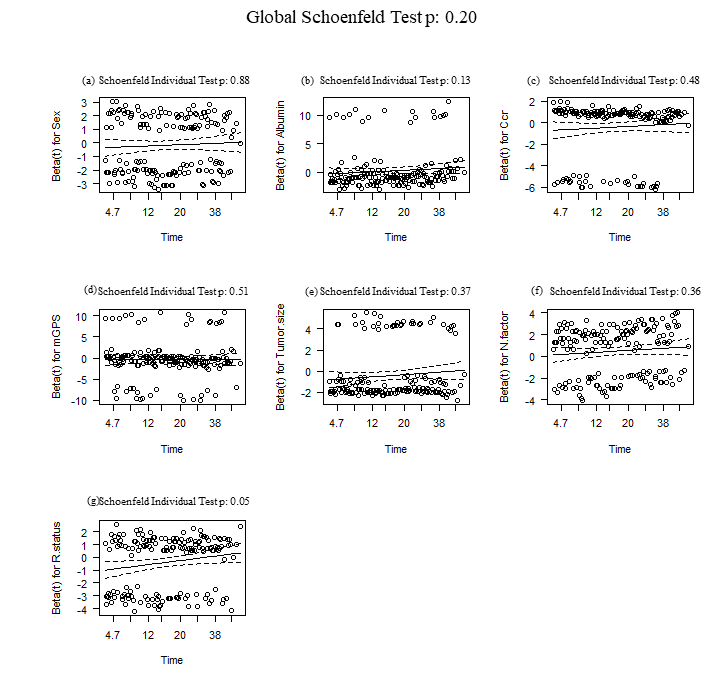

Supplement: Supplementary file 2 — Figure S2. Schoenfeld residuals used to examine the proportional hazards assumption for the variables included in Cox PH regression model in Table 3. [file CAM4-14-e70647-s006.tif]

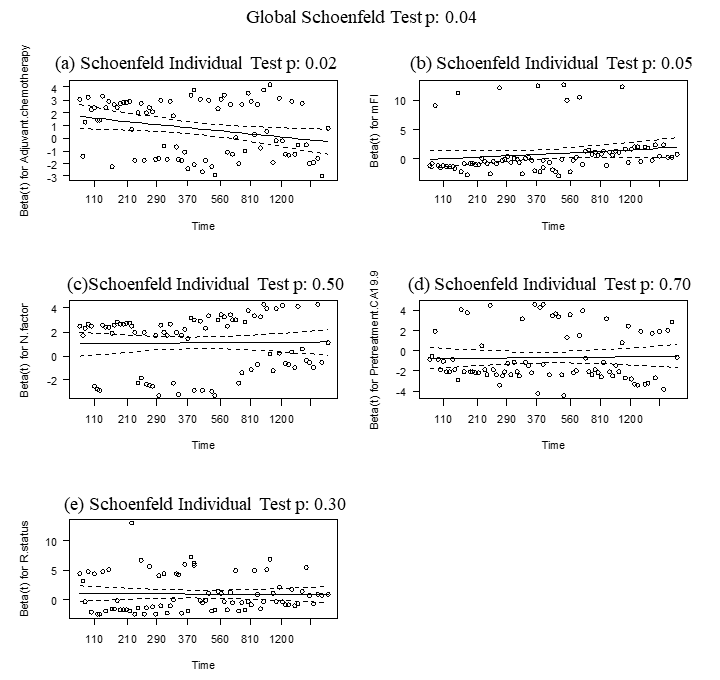

Supplement: Supplementary file 3 — Figure S3. Schoenfeld residuals used to examine the proportional hazards assumption for the variables included in Cox PH regression model in Table 4. [file CAM4-14-e70647-s007.tif]

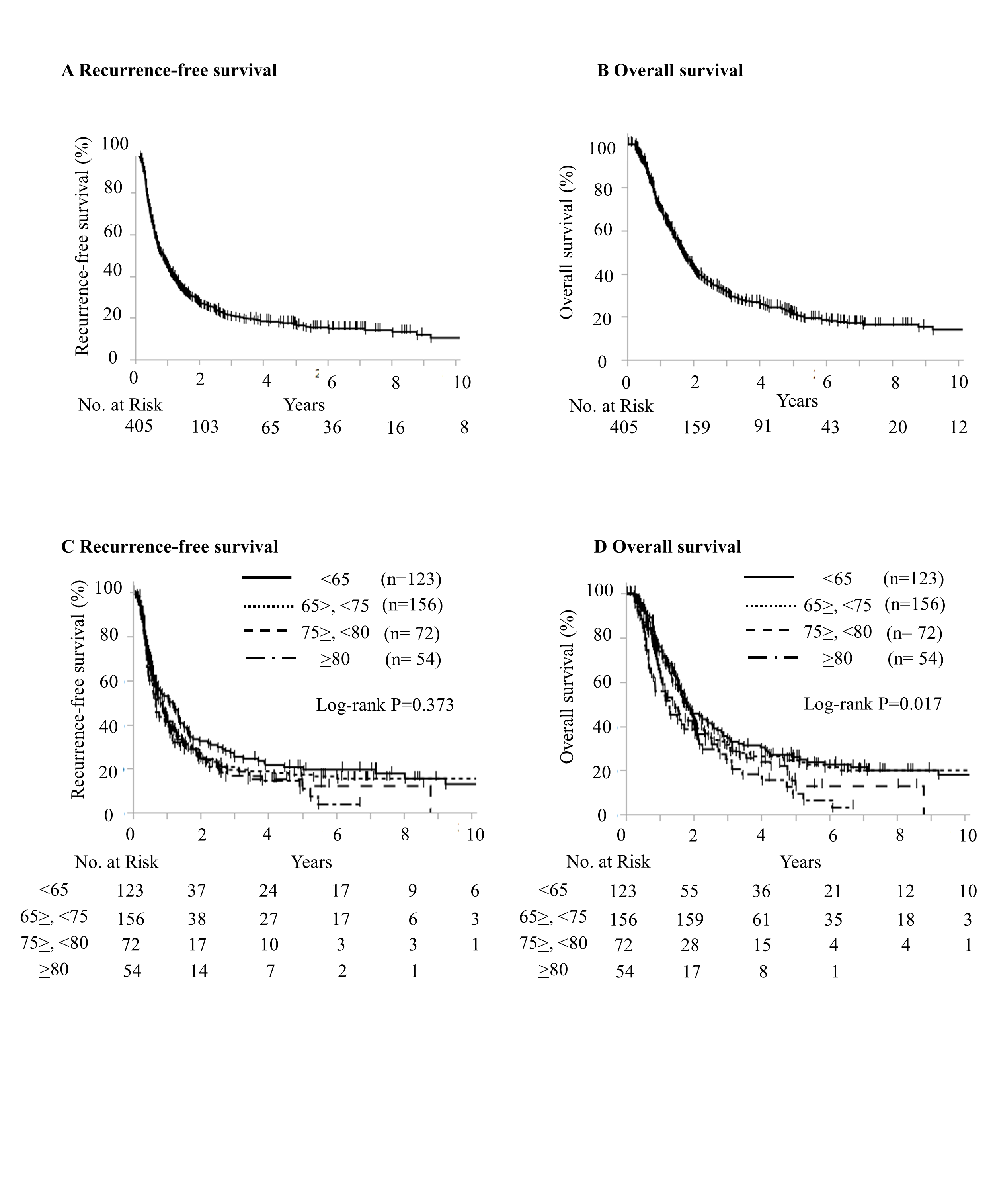

Supplement: Supplementary file 4 — Figure S4. The Kaplan–Meier curves for 323 cases excluding cases of surgery‐related mortality. Kaplan–Meier estimates of recurrence‐free survival (RFS) and overall survival (OS). (A) RFS in all patients. (B) OS in all patients. (C) RFS according to age. (D) OS by age. [file CAM4-14-e70647-s004.tif]
