# Supplementary material for: Survival Analysis of 4 Different Age Groups of Pancreatic Ductal Adenocarcinoma After Radical Resection From Retrospective Multi‐Center Analysis (YPB‐003)
Source: Cancer Med. 2025 Feb 14;14(4):e70647. doi: 10.1002/cam4.70647 (PMC11826832; doi:10.1002/cam4.70647)
Supplement: Supplementary file 5 — Table S1. Participating institutions and number of cases. [file CAM4-14-e70647-s008.docx]

| **Table S1.** Participating institutions and number of cases | | | | |
| --- | --- | --- | --- | --- |
| Institution |  | Type |  | Number of cases |
| Department of Gastroenterological, Breast and Endocrine Surgery,  Yamaguchi University Graduate School of Medicine |  | Academic center |  | 105 (25.4%) |
| Department of Surgery, Tokuyama Central Hospital |  | General hospital |  | 74 (17.9%) |
| Department of Surgery, Saiseikai Yamaguchi General Hospital |  | General hospital |  | 53 (12.8%) |
| Department of Surgery, Yamaguchi Rosai Hospital |  | General hospital |  | 49 (11.8%) |
| Department of Surgery and Clinical Science,  Yamaguchi University Graduate School of Medicine |  | Academic center |  | 36 (8.7%) |
| Department of Surgery, Kanmon Medical Center |  | General hospital |  | 33 (8.0%) |
| Department of Surgery,  Yamaguchi Prefectural Grand Medical Center |  | General hospital |  | 27 (6.5%) |
| Department of Surgery, Tsushimi Hospital |  | General hospital |  | 21 (5.1%) |
| Department of Gastroenterological Surgery,  JCHO Shimonoseki Medical Center |  | General hospital |  | 16 (3.9%) |
|  |  |  |  |  |
