# Supplementary material for: Survival Analysis of 4 Different Age Groups of Pancreatic Ductal Adenocarcinoma After Radical Resection From Retrospective Multi‐Center Analysis (YPB‐003)
Source: Cancer Med. 2025 Feb 14;14(4):e70647. doi: 10.1002/cam4.70647 (PMC11826832; doi:10.1002/cam4.70647)
Supplement: Supplementary file 6 — Table S2. 11 components of the modified frailty index. [file CAM4-14-e70647-s002.docx]

| **Table S2. 11 components of the modified frailty index** | |
| --- | --- |
| 1, | Dependent functional status |
| 2. | Pneumonia or chronic obstructive pulmonary disease |
| 3. | Diabetes mellitus |
| 4. | Prior cardiac surgery, percutaneous coronary intervention, or history of angina |
| 5. | Peripheral vascular disease or rest pain |
| 6. | Hypertension requiring medications |
| 7. | Congestive heart failure |
| 8. | Myocardial infarction |
| 9. | Transient ischemic attack or cerebrovascular accident without deficit |
| 10. | Cerebrovascular event with residual neurological deficit |
| 11. | Impaired sensorium |
|  | |
