# Supplementary material for: Survival Analysis of 4 Different Age Groups of Pancreatic Ductal Adenocarcinoma After Radical Resection From Retrospective Multi‐Center Analysis (YPB‐003)
Source: Cancer Med. 2025 Feb 14;14(4):e70647. doi: 10.1002/cam4.70647 (PMC11826832; doi:10.1002/cam4.70647)
Supplement: Supplementary file 7 — Table S3. Adjuvant Chemotherapy in Four Age Groups at Different Time Points. [file CAM4-14-e70647-s003.docx]

| **Table S3. 　Adjuvant Chemotherapy in Four Age Groups at Different Time Points** | | | | | | | |
| --- | --- | --- | --- | --- | --- | --- | --- |
|  | Adjuvant chemotherapy | All cases | Young | Pre-old | Old | Octogenarians | P value |
| **Jan.1997-Dec.2006** | | 54 | 26 | 17 | 8 | 3 |  |
|  | Gemcitabine | 8 (14.8%) | 5 (19.2%) | 2 (11.8%) | 1 (12.5%) | 0 (0%) | 0.867 |
|  | S-1 | 0 (0%) | 0 (0%) | 0 (0%) | 0 (0%) | 0 (0%) |  |
|  | Other | 4 (7.4%) | 1 (3.9%) | 2 (11.8%) | 1 (12.5%) | 0 (0%) |  |
|  | None | 42 (77.8%) | 20 (76.9%) | 13 (76.4%) | 6 (75.0%) | 3 (100%) |  |
|  | |  |  |  |  |  |  |
| **Jan.2007-Dec.2012** | | 133 | 42 | 54 | 18 | 19 |  |
|  | Gemcitabine | 81 (60.9%) | 27 (64.3%) | 38 (70.4%) | 8 (44.4%) | 8 (42.1%) |  |
|  | S-1 | 15 (11.3%) | 5 (11.9%) | 4 (7.4%) | 2 (11.1%) | 4 (21.1%) | 0.209 |
|  | Other | 4 (3.0%) | 2 (4.8%) | 2 (3.7%) | 0 (0%) | 0 (0%) |  |
|  | None | 33 (24.8%) | 8 (19.1%) | 10 (18.5%) | 8 (44.4%) | 7 (36.8%) |  |
|  |  |  |  |  |  |  |  |
| **Jan.2013-Dec.2016** | | 227 | 58 | 86 | 47 | 36 |  |
|  | Gemcitabine | 32 (14.1%) | 14 (24,1%) | 13 (15.1%) | 4 (8.5%) | 1 (2.8%) |  |
|  | S-1 | 108 (47.6%) | 32 (55.2%) | 46 (53.5%) | 21 (44.7%)) | 9 (25.0%) | <0.001 |
|  | Other | 9 (4.0%) | 1 (1.7%) | 4 (4.7%) | 3 (6.4%) | 1 (2.8%) |  |
|  | None | 78 (34.4%) | 11 (19.0%) | 23 (26.7%) | 19 (40.4%) | 25 (69.4%) |  |
