# Supplementary material for: Survival Analysis of 4 Different Age Groups of Pancreatic Ductal Adenocarcinoma After Radical Resection From Retrospective Multi‐Center Analysis (YPB‐003)
Source: Cancer Med. 2025 Feb 14;14(4):e70647. doi: 10.1002/cam4.70647 (PMC11826832; doi:10.1002/cam4.70647)
Supplement: Supplementary file 8 — Table S4. Modified frailty index and organ function. [file CAM4-14-e70647-s001.docx]

| **Table S4. modified Frailty Index and organ function.** | | | | | | | |
| --- | --- | --- | --- | --- | --- | --- | --- |
|  | |  | Age<75, mFI<0.25 | Age<75, mFI>0.25 | Age>75, mFI<0.25 | Age>75, mFI>0.25 | P value |
| Number of patients (%) | |  | 212 (64.6%) | 10 (3.1%) | 95 (28.9%) | 11 (3.4%) |  |
| Gender | |  |  |  |  |  |  |
|  | Male |  | 109 (51.4%) | 8 (80.0%) | 53 (55.8%) | 6 (54.5%) | 0.334 |
|  | Female |  | 103 (48.6%) | 2 (20.0%) | 42 (44.2%) | 5 (45.5%) |  |
| Performance Status | |  |  |  |  |  |  |
|  | 0 |  | 156 (73.6%) | 6 (60.0%) | 42 (44.2%) | 3 (27.3%) | <0.0001 |
|  | 1 |  | 52 (24.5%) | 4 (40.0%) | 50 (52.6%) | 8 (72.7%) |  |
|  | 2 |  | 4 (1.9%) | 0 (0%) | 3 (3.2%) | 0 (0%) |  |
| Body mass index in kg/m2, n (%) | |  |  |  |  |  |  |
|  | <18.5 |  | 38 (17.9%) | 1 (10.0%) | 20 (21.1%) | 4 (36.4%) | 0.462 |
|  | 18.5–24.9 |  | 147 (69.3%) | 9 (90.0%) | 64 (67.4%) | 5 (45.5%) |  |
|  | >25 |  | 27 (12.8%) | 0 (0%) | 11 (11.6%) | 2 (18.2%) |  |
| Preoperative Albumin level (g/dl) | |  |  |  |  |  |  |
|  | <3.5 |  | 38 (17.9%) | 20 (20.0%) | 28 (29.5%) | 1 (9.1%) | 0.099 |
|  | >3.5 |  | 174 (82.1%) | 8 (80.0%) | 67 (70.5%) | 10 (90.9%) |  |
| Creatinine Clearance (Cockcroft-Gault Formula) (mL/min) | | |  |  |  |  |  |
|  | <60 |  | 37 (17.5%) | 2 (20.0%) | 58 (61.1%) | 10 (90.9%) | <0.0001 |
|  | >60 |  | 175 (82.5%) | 8 (80.0%) | 37 (38.9%) | 1 (9.1%) |  |
| Surgical complication | |  |  |  |  |  |  |
|  | CD<III |  | 182 (85.9%) | 10 (100%) | 80 (84.2%) | 9 (81.8%) | 0.577 |
|  | CD>III |  | 30 (14.1%) | 0 (0%) | 15 (15.8%) | 2 (18.2%) |  |
| In-hospital mortality | |  |  |  |  |  |  |
|  | No |  | 210 (99.1%) | 10 (100%) | 92 (96.8%) | 11 (100%) | 0.477 |
|  | Yes |  | 2 (0.9%) | 0 (0%) | 3 (3.2%) | 0 (0%) |  |
| Adjuvant chemotherapy | |  |  |  |  |  |  |
|  | No |  | 56 (26.4%) | 4 (40.0%) | 51 (53.7%) | 8 (72.7%) | <0.0001 |
|  | Yes |  | 156 (73.6%) | 6 (60.0%) | 44 (46.3%) | 3 (27.3%) |  |
| Relative dose intensity of adjuvant chemotherapy ,median (range), % | | | |  |  |  |  |
|  |  |  | 68.8 (15.9–100) | 78.7 (41.2-100) | 59.4 (9.0–100) | 51.7 (30.8–62.5) | 0.015 |
| mFI, modified Frailty Index; CD, Clavien-Dindo classification | | | | | | | |
